# The complete chloroplast genome sequence of *Ulmus elongata* (Ulmaceae)

**DOI:** 10.1080/23802359.2020.1715280

**Published:** 2020-01-24

**Authors:** Huang Yinran, Huang Xiaoxu, Feng Shuxiang, Yan Shufang, Li Yongtan, Liu Yichao

**Affiliations:** a Hebei Academic of Forestry and Grassland, Shijiazhuang, China;; b Hebei Engineering Research Center for Trees Varieties, Shijiazhuang, China;; c Hebei Agricultural University, Baoding, China

**Keywords:** Chloroplast genome, *Ulmus elongata*, Ulmaceae

## Abstract

*Ulmus elongata* is a species of Sect. Chaetoptelea (Liemb.) S chneid in Ulmaceae, and it is an endangered wild plant listed in the second class of the Protected Plants in China. The complete chloroplast genome (cp) of *U. elongata* was reported in this study. The result showed that the cp genome was 159,230 bp in length including a large single-copy (LSC) 87,718 bp and a small single-copy (SSC) 18,690 bp, which were separated by two inverted repeats (IRs) of 26,411 bp with the typical quadripartite structure, respectively. The genome encoded 132 genes, including 87 protein-coding genes, 37 tRNA genes, and eight rRNA genes. The GC content was 35.57%. Chloroplast sequences were used for constructing phylogenetic tree to determine the evolutionary status of *U. elongata*. The maximum-likelihood phylogenetic analysis showed that *U. elongata* was clustered with five other *Ulmus* species, and the relationship between *Ulmus* and *Zelkova* was closest. The success of cp genome assembly of *U. elongata* has laid a foundation for the study of chloroplast molecular biology and can effectively promote the study of genetic breeding and molecular evolution of *U. elongata*.

Chloroplasts are important organelles in photosynthesis (Li et al. 2018; Santos and Almeida 2019; Yan et al. 2019). Compared with the nuclear genome, the chloroplast genome size is smaller, with very conservative gene composition, gene sequence and gene type, lower nucleotide replacement frequency, and the cp genomes are inherited uniparentally (maternally in most angiosperms plants) (Abdullaha et al. 2019; Song et al. 2019; Zhang et al. 2019), which is widely used in studying phylogeny of plant species. The plants of *Ulmus* in the family Ulmaceae are mostly positive and sun-loving plant, which are characterized by drought tolerance, cold tolerance, barren tolerance, pruning tolerance, fast growth, strong adaptability, and other characteristics (Zhang 2016; Shi et al. 2017). *Ulmus elongata*, the Sect. Chaetoptelea (Liemb.) S chneid of *Ulmus*, is a unique rare species with a limited population in China (Chen and Zeng 2017; Luo et al. 2018) and has been listed in the IUCN Red List of Endangered Species (endangered EN level) and the first batch of National Key Protected Wild Plants (listII level).

The experimental material (stored in herbarium of Hebei Academic of Forestry and Grassland, File number: HAFG22U340) donated by Mr. Fang Teng, the general manager of Jiashan Lige Ecological Technology Co., LTD, was now planted in the Hebei Academic of Forestry and Grassland, Shijiazhuang, China (114°28′12″E, 38°08′23″N). In July 2019, young leaves of *U. elongata* were stored in liquid nitrogen and sent to Beijing Zhongxing Bomai Technology Co., LTD for sequencing, assembly, and annotation of the total chloroplast genome. Plant DNA extraction kit (TIANGEN, Beijing) was used to extract the total DNA of fresh young leaves. Illumina NovaSep platform was used for sequencing after meeting the sequencing requirements. The original data were filtered to obtain high-quality data, and SOAPdenovo (Luo et al. 2012) software was used to assemble the data. OGDRAW software (Lohse et al. 2013) was used to draw the physical map of chloroplast genome (uploaded to NCBI with the number of MN720267).

Similar to other higher plants, *U. elongata* had a typical quadripartite structure consisting of two single-copy regions (LSC of 87718 bp and SSC of 18690 bp) and a pair of IRs regions of 26,411 bp. The plastome sequence of *U. elongata* was 159,230 bp. The guanine-cytosine (GC) content was 35.57%. The genome contained 132 genes, including 87 protein-coding genes, 37 tRNA genes, and eight rRNA genes. The phylogenetic tree was constructed based on the complete chloroplast sequences of *U. elongate* and 16 other plant species available in the NCBI database (including five *Ulmus* species, one *Pteroceltis* species, one *Celtis* species, one *Zelkova* species, two *Morus* species, two *Quercus* species, three *Populus* species, and a outgroup *Arabidopsis*) using raxmlGUI version 1.5 b (https://sourceforge.net/projects/raxmlgui/) with 1000 bootstrap replicates (Silvestro and Michalak 2012). The result showed that *U. elongata* was clustered with five other *Ulmus*, and the relationship between *Ulmus* and *Zelkova* was closest (Figure 1).

**Figure 1.**
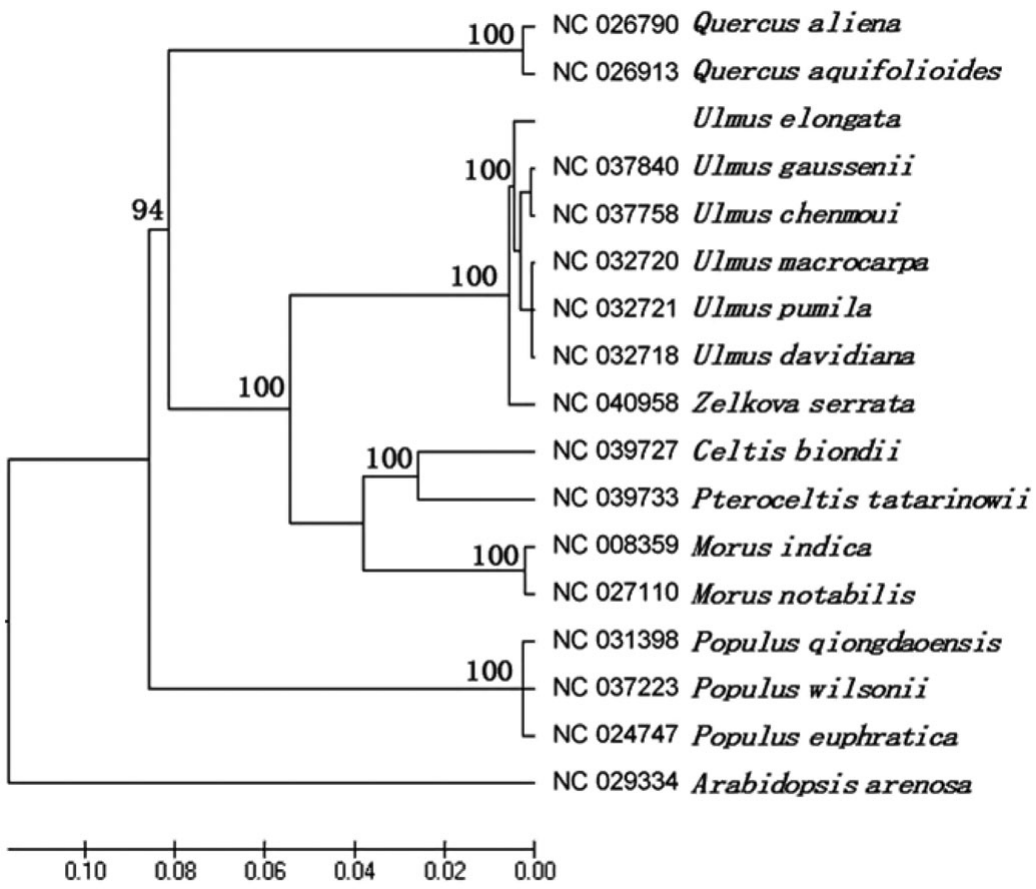
Maximum-likelihood phylogenetic tree based on 17 selected plants chloroplast genome sequences..
